# Imaging Neuronal Activity in the Optic Tectum of Late Stage Larval Zebrafish

**DOI:** 10.3390/jdb6010006

**Published:** 2018-03-09

**Authors:** Katharina Bergmann, Paola Meza Santoscoy, Konstantinos Lygdas, Yulia Nikolaeva, Ryan B. MacDonald, Vincent T. Cunliffe, Anton Nikolaev

**Affiliations:** 1 Department of Biomedical Science, The University of Sheffield, Western Bank, Sheffield S10 2TN, UK; kbergmann1@sheffield.ac.uk (K.B.); paola.mezasantoscoy@ucalgary.ca (P.M.S.); klygdas1@sheffield.ac.uk (K.L.); y.nikolaeva@sheffield.ac.uk (Y.N.); 2 Department of Infection, Immunity and Cardiovascular Disease, The University of Sheffield, Western Bank Sheffield, S10 2TN, UK

**Keywords:** zebrafish, calcium imaging, neuronal activity

## Abstract

The zebrafish is an established model to study the development and function of visual neuronal circuits in vivo, largely due to their optical accessibility at embryonic and larval stages. In the past decade multiple experimental paradigms have been developed to study visually-driven behaviours, particularly those regulated by the optic tectum, the main visual centre in lower vertebrates. With few exceptions these techniques are limited to young larvae (7–9 days post-fertilisation, dpf). However, many forms of visually-driven behaviour, such as shoaling, emerge at later developmental stages. Consequently, there is a need for an experimental paradigm to image the visual system in zebrafish larvae beyond 9 dpf. Here, we show that using *NBT:GCaMP3* line allows for imaging neuronal activity in the optic tectum in late stage larvae until at least 21 dpf. Utilising this line, we have characterised the receptive field properties of tectal neurons of the 2–3 weeks old fish in the cell bodies and the neuropil. *The NBT:GCaMP3* line provides a complementary approach and additional opportunities to study neuronal activity in late stage zebrafish larvae.

## 1. Introduction

The zebrafish has become an important model organism to study the development and organisation of neuronal circuits involved in various forms of behaviour. Many experimental paradigms combining behavioural assays with imaging of neuronal activity have been developed and applied to study the tuning properties of visual neurons in the retina, optic tectum, pretectum, and habenula [1,2,3,4,5,6,7].

The transparent nature of larval zebrafish enables optical imaging of the entire zebrafish brain up to the age of 8 days post-fertilisation (dpf), which is shortly after simple forms of visually-driven behaviour and independent feeding have started to emerge [8]. However, other visually-driven behaviours, like shoaling, pattern discrimination, and novel object recognition, appear later in development (e.g., 10–30 dpf, [9,10,11]). Thus, a robust method for imaging neuronal activity in older fish is needed.

A disadvantage of the commonly used pan-neuronal *huc* promoter is that the expression of neuronal activity reporters (e.g., GCaMPs) decreases after 6–7 dpf [12]. To overcome this issue, one can either use GCaMP3-H2B fusion to target the calcium reporter expression to the nucleus [12] or use synthetic calcium indicators injected in the brain area of interest [13]. While both approaches have proven useful they have limitations. Targeting the calcium reporter to the nucleus does not allow for imaging activity at individual synapses, a crucial component of multiple forms of neuronal computations [3,4,14,15,16]. Additionally, targeting synthetic calcium indicators to specific neuronal types or cellular compartments is not straightforward.

Using more recent and brighter forms of GCaMPs (e.g., GCAMP5 and GCaMP6) has allowed for the imaging period to be extended beyond 14 dpf, particularly in the habenula [17,18,19]. However, to the best of our knowledge imaging from the optic tectum has not been reported for late stage larvae. The optic tectum is of particular interest, as it represents the central visual processing area in lower vertebrates. It is involved in a number of visual behaviours, such as those that rely on a detailed map of visual space (e.g., photo taxis [20], prey detection and capture [21,22] and escape responses [23,24]). The tectal neurons encode fundamental information about objects in the visual space, such as direction of motion and global orientation [3,15,25], and object size [1,4]. Thus, having the ability to measure neural activity in this area beyond the current timelines will allow for the study of many important and not well-understood behavioural processes.

Here we report an experimental paradigm that allows robust imaging of neuronal activity in the optic tectum of late-stage larvae up to the age of 21 dpf. We have generated a transgenic line driving pan-neuronal expression of GCaMP3 under the control of the neural beta-tubulin promoter (*NBT*, [26]). The reporter is expressed throughout the brain, including the optic tectum and retina with expression levels remaining high for at least three weeks post-fertilisation. Using this line, we have characterised the receptive fields of tectal neurons in 2–3 weeks old larvae. The experimental approach described here provides an alternative to commonly used *huc*-driven lines and an opportunity to better understand the development and maturation of neuronal circuits in the optic tectum.

## 2. Materials and Methods

All experimental procedures were performed according to UK Home Office regulations (Animals (Scientific Procedures) Act 1986) and approved by the Ethical Review Committee at the University of Sheffield.

### 2.1. Animals and Husbandry

Zebrafish were bred using a standard breeding over marbles procedure described on https://zfin.org/zf_info/zfbook/chapt2/2.6.html. The embryos were raised in standard E3 solution without methylene blue, at 28 °C and on a 14/10 light/dark cycle. At 5 dpf, the fish were moved to a Tecniplast system (Tecniplast S.p.A., Buguggiate, Italy) and fed twice a day, including the day of the experiment [2]. No more than 40 fish were housed per tank.

### 2.2. Generation of the NBT:GCaMP3 Transgenic Line

**Figure 1:**
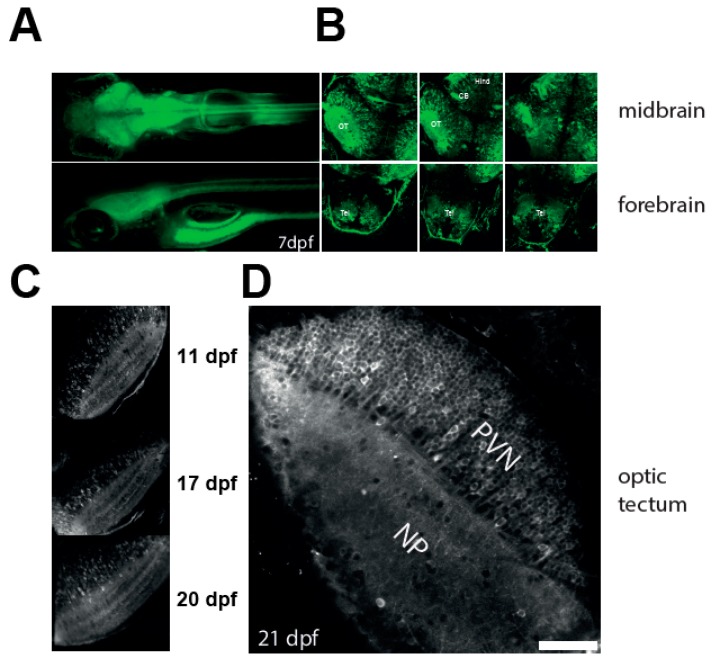
Characterisation of the NBT:GCaMP3 line. (**A**) Wide fluorescence imaging of 7 dpf larvae expressing GCaMP3 under control of *NBT* promoter. Top—dorsal view. Bottom—lateral view. (**B**) Confocal imaging of 7 dpf *NBT*:GCaMP3 larvae at three different depths. Note that GCaMP3 fluorescence is evident in all major areas of the brain. CB: cerebellum, Hind: hindbrain, OT: optic tectum, Tel: telencephalon. (**C**–**D**) GCaMP3 expression is robust in fish aged 10 to 21 dpf. Shown are representative images of the optic tectum at 11, 17, 20 (**C**), and 21 dpf (**D**). Note that majority of tectal periventricular cells bodies are labelled with GCaMP3 (**D**). NP: neuropil, PVN: periventricular neurons. Scale bar—50 µm.

A transgenic zebrafish line, expressing the calcium reporter GCaMP3 [27] under the control of the Xenopus neuronal beta-tubulin (*NBT*) promoter (Figure 1) [26], was generated by using the Tol2 transgenesis system [28,29,30,31]. An *NBT:GCaMP3* cassette was subcloned into a miniTol2 vector and injected into one-cell stage zebrafish embryos of the *nacre* line [32], along with Tol2 transposase mRNA. Injected embryos exhibiting mosaic expression of GCaMP3 in the CNS at 2 dpf were identified using a Leica MZ-16 fluorescence dissecting microscope (Leica Microsystems, Wetzlar, Germany). The embryos were then raised to adulthood and crossed with *nacre* fish at 3 months of age to identify founders exhibiting germline transmission of *NBT:GCaMP3*. Stable F1 families of transgenic fish were established, from which non-mosaic progeny expressing *NBT-GCaMP3* throughout the nervous system were obtained.

### 2.3. Imaging and Visual Stimulation

**Figure 2:**
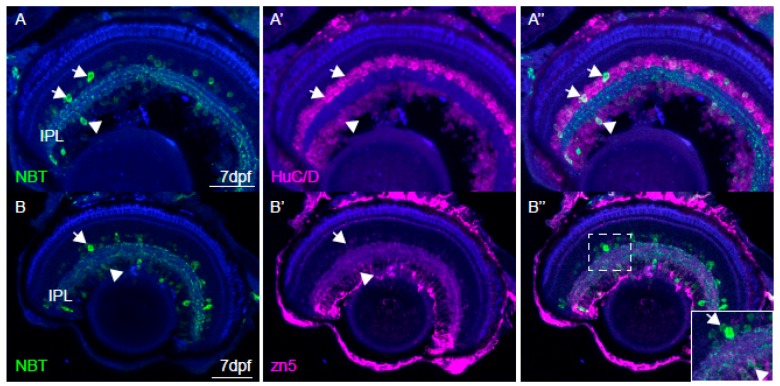

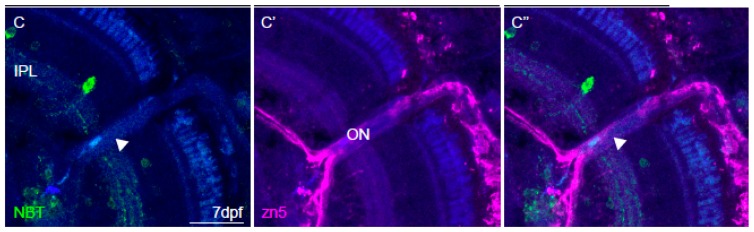
Characterisation of *NBT*:GCaMP3 expression in the eye of 7 dpf fish. (**A**) The *NBT:GCaMP3* transgene product, visualised with an anti-GFP antibody (green), sparsely labels HuC/D positive neurons (magenta) that project to the synaptic inner plexiform layer (IPL). Amacrine cells are labelled in the inner nuclear layer (arrows) and retinal ganglion cells are labelled in the ganglion cell layer (arrowheads). (**B**) Anti-GFP antibodies label only a small number of zn5-positive retinal ganglion cells (arrowhead). GFP-positive amacrine cells in the inner nuclear layer are zn5-negative (arrow). (**C**) The *NBT:GCaMP3 transgene* is expressed in a small number of retinal ganglion cell axons in the optic nerve (ON, arrowhead). The blue label is a counterstain. Scale bars in A = 40 µm, B = 50 µm, C = 25 µm.

Imaging was performed as described in [15], but modified to allow imaging of larger, i.e., 14–21 dpf larvae: (i) Because of their larger size (around 8 mm at 18 dpf), a higher concentration of low-melting point agarose was used (3.5%) to immobilise the fish. The agarose covering the gills was carefully removed. The general state of the fish was assessed by monitoring of the heartbeat before and after the experiment. (ii) To increase oxygen absorption through the skin during the experiment, the imaging solution was oxygenated prior to the experiment. The imaging solution was prepared as follows: 500mL of aquarium water was filtered (Grade 1, Whatman filter, GE Healthcare UK Limited, Little Chalfont, UK) and buffered in 1.2 mM NaH_2_PO_4_ and 23 mM NaHCO_3_ (Sigma-Aldrich, Sigma-Aldrich, St. Louis, MO, USA). The solution was then aerated with a mix of 95% O_2_ and 5% CO_2_ for at least 30 min (Supplementary Figure S1A, left) in a standard glass bottle (500 mL, Cole-Parmer, Stone, UK). The O_2_/CO_2_ mix was supplied via a small plastic tube fitted to the regulator valve of the gas bottle (Supplementary Figure S1A, right). The outer dimensions of the imaging chamber were 90 (L) × 70 (W) × 15 mm (H) and a total amount of 64 mL of oxygenated imaging solution was added to the chamber after the fish was mounted (Supplementary Figure S1B). The agarose for immobilising the fish was dissolved in oxygenated solution. No oxygenation was applied during the experiment. These modifications allowed us to image 14–21 dpf fish for up to 3 h. (iii) Due to the limited working distance of the microscope, the screen size was 90 mm (W) × 15 mm (H) (148 × 60 degrees). Fish watched the screen with the right eye, with an eye-to-screen-distance of 1.3 cm. Imaging of the eye was performed on 10 dpf *casper* mutants expressing *NBT:GCaMP3*, which were additionally treated with 200 mM 1-phenyl-2-thiourea (PTU) from 1–10 dpf to avoid pigment formation (older fish were not studied, due to the detrimental effect of PTU on the health of zebrafish). Fish were mounted sideways and immobilised in 1.5% agarose for confocal imaging. To characterise the expression of GCaMP3 in the eye at later stages, we performed sectioning of the eye of 7 dpf and 21 dpf (Figure 2C).

Visual stimuli were generated using custom-written code for Matlab (MathWorks, Natick, MA, USA) with the Psychophysics Toolbox [33,34,35] using an Optoma PK320 projector connected to a Linux laptop. Imaging of neural activity was performed using a FV1000 confocal microscope (Olympus, Tokyo, Japan) at the Wolfson imaging facility (The University of Sheffield), fitted with a 40× (NA 0.8) LUMPlan objective (Olympus, Tokyo, Japan). Sampling frequency was 2.3 Hz.

### 2.4. Image Analysis

**Figure 3:**
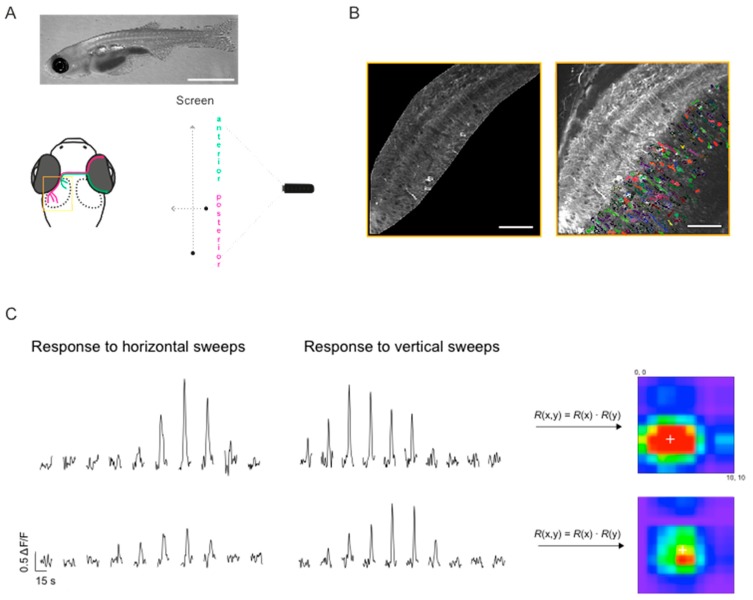

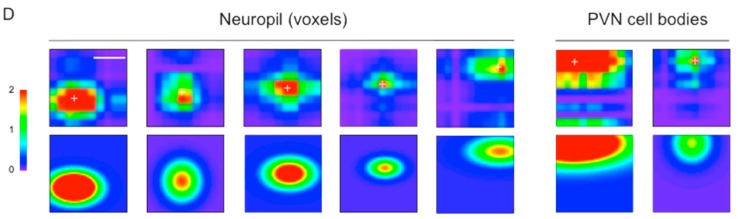
Mapping receptive fields in the optic tectum with small spots. (**A**) Schematic diagram of the experimental setup for visual stimulation, also illustrating the retinotopic organisation of visual input to the optic tectum: Retinal ganglion cells located in the anterior part (magenta) of the retina, convey information about the posterior part of the visual field and project to the posterior part of the optic tectum and vice versa. Yellow box shows the region imaged in (**B**). Top—representative image of an 18 dpf larvae (scale bar: 2 mm). (**B**) Dorsal view of one tectal hemisphere of a transgenic zebrafish larvae (18 dpf) expressing GCaMP3 in both the tectal neuropil (left panel) and cell bodies of PVN neurons (right panel). Confocal time series were analysed either on a voxel-wise basis (neuropil) or on a region of interest (ROI) basis (cell bodies). Cell bodies were randomly colour-coded for better visualisation. (**C**) Representative ΔF/F traces for two voxels in the neuropil, in response to a small spot moving horizontally and vertically across a 10-by-10 grid on the screen. The stimuli were presented in a pseudo-random order; the response traces shown here were manually sorted for presentation purposes. Responses to vertical and horizontal sweeps were multiplied for each location resulting in the 2D receptive fields (RFs) shown on the right. White cross indicates RF centre as determined by parametric fitting with a bivariate Gaussian distribution. (**D**) Top: Examples of spatial RFs in the tectal neuropil and cell bodies of PVN neurons, colour-coded to show response amplitudes for each point on a 10-by-10 grid. White cross indicates RF centre as determined by parametric fitting with a bivariate Gaussian distribution. Bottom: 2D RFs fitted with a bivariate Gaussian distribution. Scale bars: 50 μm (fluorescence images), 20 degrees (RFs).

Confocal time-series were analysed using SARFIA [36] and custom-written scripts for Igor Pro (WaveMetrics, Lake Oswego, OR, USA). Prior to further analysis, images were registered (TurboReg for ImageJ, [37]) to remove motion artefacts, and filtered to improve the signal-to-noise ratio (only for neuropil: median filter, kernel size = 1; Gaussian smoothing, kernel size = 2). Cell bodies were identified by thresholding the average image using the Laplace operator (Figure 3B right). Data from the neuropil were analysed on a voxel-wise basis (Figure 3B left, [15]). Signal traces were normalised (∆F/F0, F0 was mean signal intensity in the 5 s before stimulation started) and averaged across three repetitions. Visually responsive traces were identified by calculating the skewness of the distribution curve for each voxel or region of interest (ROI). Traces with skewness > 1.4 (neuropil) and skewness > 0.9 (cell bodies) were regarded as responsive and included in further analysis. Threshold levels were derived empirically, and efficiently distinguished between visually responsive and unresponsive, noisy traces.

### 2.5. Mapping Receptive Field Properties

To map the spatial receptive fields (RFs) of tectal neurons, we used a small black spot (4.88 degrees in diameter), sweeping across a 10 × 10 grid in two cardinal directions (caudal-to-rostral and top-to-bottom, Figure 3A). A similar approach has been successfully used in the past to map the spatial RFs of tectal cells in zebrafish [38,39], where it proved to be faster and more efficient than white noise [38]. In brief, the responses to horizontal and vertical sweeps (Figure 3C) were multiplied for each point on a 10 × 10 grid. The resulting two-dimensional RF was then fitted with a bivariate Gaussian distribution and the RF width and height were defined as 4σ of the Gaussian along the respective axis (Figure 3C right). All data are shown as mean ± SEM.

### 2.6. Immunohistochemistry and Imaging

Cryosections were taken from paraformaldehyde fixed tissue at 12 μm thickness using a Jung Frigocut cryostat (Leica Biosystems, Nussloch, Germany). Immunostaining was performed using rabbit anti-GFP (1:200, ab290, Abcam, Cambridge, UK), mouse anti-HuC/D (1:200, 16A11, Invitrogen, Carlsbad, CA, USA) and Zn5 (1:50, ZIRC, University of Oregon, Eugene, OR 97403, USA). Laser scanning confocal imaging was performed using a Leica Sp5 microscope (Leica) with a 63× silicon oil immersion objective (NA 1.3) using the LCS Leica Confocal Software (Leica Microsystems). Image analysis was performed using ImageJ and Volocity Software (Perkin Elmer, Waltham, MA, USA)

## 3. Results

### 3.1. Robust Expression of a Calcium Reporter in the Optic Tectum of NBT:GCaMP3 Fish

The commonly used *huc:GCaMP3* reporter line decreases in brightness after 7–9 dpf [12]. To overcome this problem we have generated a transgenic zebrafish line expressing the calcium reporter GCaMP3 [27] under the control of the *Xenopus* neuronal beta-tubulin (*NBT*) promoter (Materials and Methods, [26]). This promoter has been sub-cloned from *Xenopus laevis*, where it normally drives the expression of the neural-specific beta-Tubulin, and has been used for broad discrimination between neural tissue and other tissues (e.g., muscle).

Figure 1A shows low-resolution images of line at the age of 7 dpf. At this stage fluorescence was observed in all major brain areas: the forebrain, midbrain, and hindbrain. To characterise the line further, we performed confocal imaging in these areas at different depths (Figure 1B). GCaMP3 was expressed in tectal neurons (Figure 1C), as well as in the neurons of the pretectum, cerebellum, spinal cord, and forebrain areas, dorsal and ventral telencephalon, and both habenulae. To test whether expression of GCaMP3 persists at later stages of development we performed confocal imaging on *NBT:GCaMP3* fish at different ages. Figure 1C,D shows example images of the optic tectum acquired at 11, 17, 20, and 21 dpf. The fluorescence did not decrease with age, and was observed in both the tectal neuropil and the cell bodies. Thus, the *NBT:GCaMP3* line can be used to image neuronal activity in larvae beyond the age of 8–9 dpf.

In the retina sparse GCaMP3 labelling was observed in neurons projecting to the inner plexiform layer (IPL), as assessed by immunohistochemistry using anti-GFP antibodies (Figure 2A) and by in vivo confocal imaging of intact PTU-raised fish carrying the *roy* mutation (Supplementary Figure S2). These neurons are HuC/D positive in the inner nuclear layer and ganglion cell layer (Figure 2A’), identifying them as amacrine cells and/or retinal ganglion cells. A small number of cells (2.75 ± 0.9 per field of view, *n* = 10 randomly selected fields of view from five fish at 10 dpf) were labelled in the ganglion cell layer (Supplementary Figure S2). To test whether these cells were ganglion cells or misplaced amacrine cells, we performed immunohistochemistry, in which retinal ganglion cells were labelled with anti-Zn5 antibodies and GCaMP3 with anti-GFP antibodies (Figure 2B,2B’ respectively). Most Zn5 positive cells were not labelled by anti-GFP antibodies. This was particularly evident in the optic nerve (Figure 2C,2C’), where only a small portion of optic nerve axons is labelled by GFP antibodies. Thus, we presume that the GCaMP3 signal detected in the tectal neuropil originates from the postsynaptic neurons or non-retinal projections from other brain areas. Similar results were obtained using 21 dpf fish (Supplementary Figure S3). This is in contrast with lines using another pan-neuronal promoter, *huc*, which drives expression in all retinal ganglion cells [40]. Although individual retinal ganglion cells may exhibit expression of GCaMP3 (Figure 2B and Supplementary Figure S3), weak expression in these cells creates an opportunity for simultaneous imaging of presynaptic retinal input and postsynaptic tectal neurons in the neuropil, which would provide valuable information to understand tectal circuitry.

### 3.2. Imaging Neuronal Activity in the Optic Tectum of 3 Weeks Old Larvae

**Figure 4:**
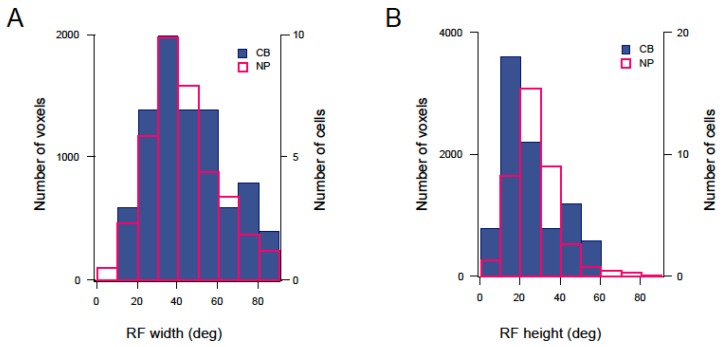

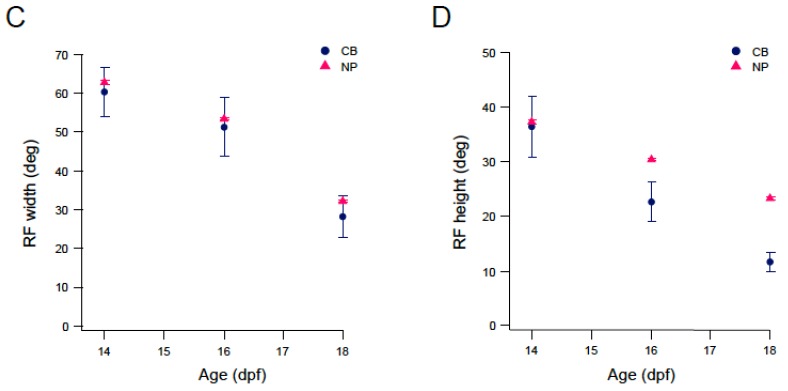
Spatial receptive field sizes in the optic tectum of 14, 16, and 18 dpf larvae (**A**,**B**) Distribution of RF width (**A**) and height (**B**) in the neuropil (magenta) and PVN cell bodies (blue). RF size was determined by parametric fitting of a bivariate Gaussian distribution and defined as 4σ. (**C**) Average spatial RF width at 14, 16, and 18 dpf. (**D**) Average spatial RF height at 14, 16, and 18 dpf. (NP: *n* = 7790 voxels from 6 fish; CB: *n* = 48 cells from 5 fish).

**Figure 5:**
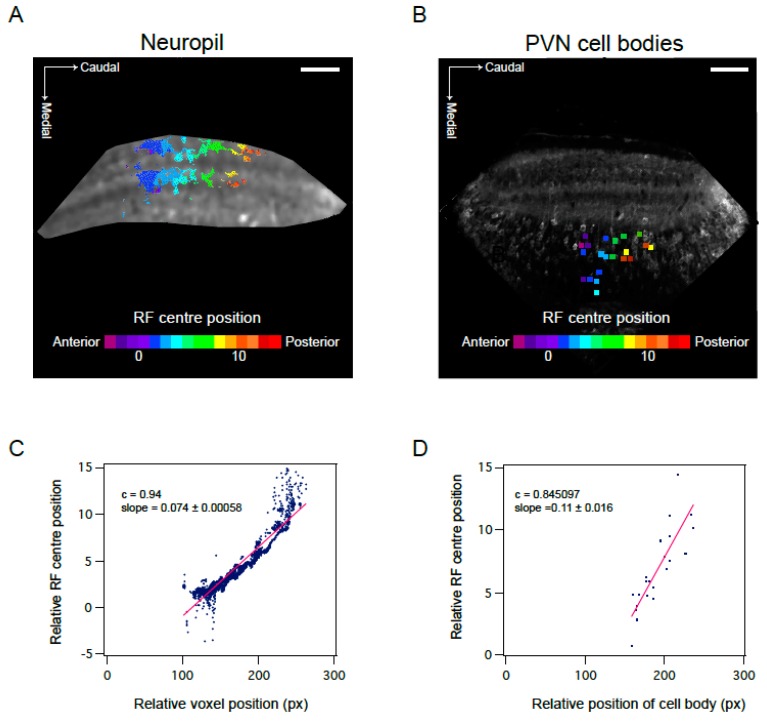
Visuotopic organisation of RF centres in the optic tectum. (**A**,**B**) Position of RF centre as determined by parametric fitting of a bivariate Gaussian distribution along anterior—posterior axis shows visuotopic organisation in the neuropil (**A**) and PVN cell bodies (**B**). Representative examples of one field of view. (**C**,**D**) Visuotopic correlation illustrated as regression fit to RF centre versus relative position of voxel (**C**) or cell body (**D**) within the optic tectum (in pixels). Data corresponds to fields of view shown in A and B. c calculated as Pearson’s correlation coefficient. Scale bar: 50 µm.

Next we tested whether the *NBT:GCaMP3* line can be used to study neuronal activity in the optic tectum (Figure 3, Figure 4 and Figure 5). As immobilisation in agarose may prevent sufficient oxygenation of large larvae, we developed a technique where the fish was placed in a buffered and oxygenated solution during imaging, thereby allowing more oxygen to be absorbed through the skin (see Materials and Methods). Using the *NBT:GCaMP3* line and oxygenation of the imaging solution we were able to image neuronal activity in 14–21 dpf old fish (Figure 3 and Figure 4) for up to 3 h.

### 3.3. Mapping RF Properties in the Tectal Neuropil and the Cell Bodies

We then used our experimental paradigm to characterise the spatial receptive fields (RFs) of tectal neurons in 2–3 weeks old larvae using sweeping spots, moving in vertical and horizontal directions (Materials and Methods and Figure 3A). About 5–20% of cells per field of view exhibited robust responses to the spots at different positions (Figure 3C). Manual inspection revealed a variety of RFs, differing in size, shape, and position of the centre, both in the neuropil (Figure 3D, left) and the cell bodies of the periventricular neurons (Figure 3D, right). Most RFs could be described as having a round or oblong shape, and a clearly defined single centre. The majority of oblong RFs were elongated in horizontal direction (average x/y ratio was 1.93 ± 0.07 for neuropil (7790 voxels, 6 fish) and 3.11 ± 0.47 for cell bodies (48 cells, 5 fish)).

Generally, RF sizes in the neuropil were similar to those in the cell bodies. The majority of RFs were between 10 and 90 degrees in width, and 10 and 50 degrees in height. Mean RF sizes (4σ) were 44.94 ± 0.24 degrees (width) and 28.42 ± 0.16 degrees (height) for the neuropil (*n* = 7790 voxels 6 fish, Figure 4A,B, magenta bars) and 53.02 ± 4.45 degrees (width) and 28.73 ± 3.59 degrees (height) for cell bodies (*n* = 48 cells, 5 fish, Figure 4A,B, blue bars). As the stimuli were only presented in a small portion of the visual field (48.88 × 48.88 degrees), many of the RFs were located at the borders of the grid, and/or only a portion of the RF was mapped. To test whether fitting these RFs with a bivariate Gaussian distribution (Materials and Methods) still resulted in a correct estimate of RF size, we compared the size distribution of all RFs in the neuropil with the distribution of a subset of RFs, whose centre was located in the middle of the grid. RF size distribution, as well as mean RF size values for both groups were similar, and we therefore assumed that the result of the Gaussian fit correctly predicted RF size, even for RFs which were partly located outside the grid (NP, mean RF width: 44.94 ± 0.24 (all), 42.41 ± 0.29 (centre-only); mean RF height: 28.42 ± 0.16 (all), 24.95 ± 0.19 (centre-only)).

To date, tectal RF sizes have been reported for early larvae (5 to 9 dpf) and adult zebrafish [38,39]. However, between 10 and 20 dpf, fish start to exhibit more complex visual behaviours. For example, they start to reliably shoal with conspecifics [10,41]. They also demonstrate a reliable exploratory behaviour and can memorise 3D objects for many hours [9]. Whether these changes are reflected in the RF properties in the optic tectum is unknown. Using the *NBT:GCaMP3* line, we imaged neuronal activity in 2–3 weeks old larvae, and studied how tectal RF sizes change with age. Interestingly, our results show a marked decrease in RF size from 14 to 18 dpf. Figure 4C,D illustrate the average spatial RF size from six (five for cell bodies) individual fish aged 14, 16, and 18 dpf. Both width and height of the RFs decreased with age, suggesting a continued refinement of spatial RFs (e.g., in the neuropil, RF width was 62.58 ± 0.53 at 14 dpf, and 32.2 ± 0.2 at 18 dpf, average of two individual fish).

As expected, both cell bodies and the neuropil exhibited retinotopic organisation: the position of the RF centre correlated with the relative location of the cell or ROI within the optic tectum. Cells and ROIs located at the rostral end of the tectum responded to stimuli in the anterior portion of the visual field, whereas cells and ROIs at the caudal end responded to stimuli in the posterior part (Figure 5). This correlation was strong for both cell bodies (Figure 5B,D) and ROIs in the neuropil (Figure 5A,C) in all fish (NP: mean correlation coefficient (Pearson’s correlation coefficient, PCC) for all fish = 0.83 ± 0.023, *n* = 7790 voxels in 6 fish; CB: mean correlation coefficient (PCCs) for all fish = 0.85 ± 0.033, *n* = 48 cells from 5 fish). A corresponding relationship between the top/bottom visual axis and the dorsoventral axis was observed when imaging at different depths. ROI position in relation to neuropil layer had no effect on the position of the RF centre (Figure 5A).

### 3.4. Imaging of Neuronal Activity in Other Brain Areas

**Figure 6:**
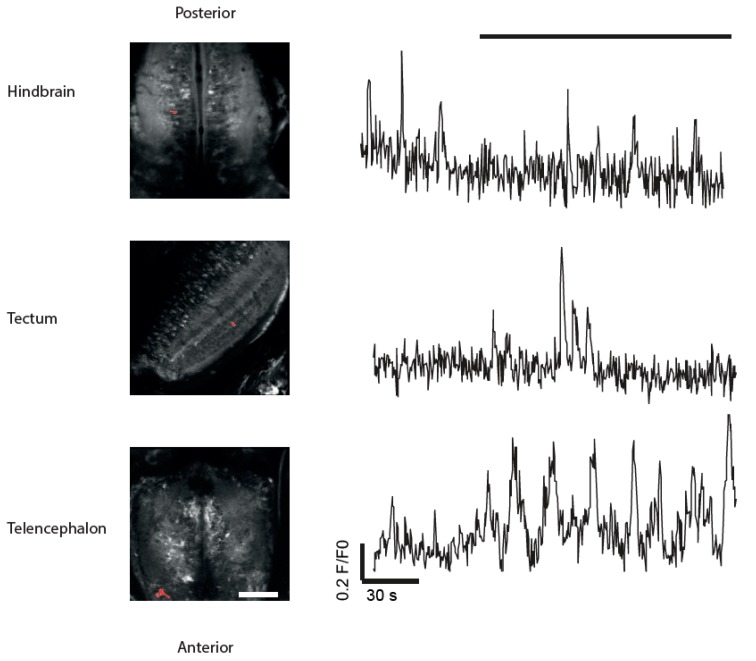
Spontaneous (independent of visual stimulation) activity can be observed in brain areas other than optic tectum using the *NBT:GCaMP3* line. Left: Representative images of hindbrain, tectum and the telencephalon of 14 dpf fish used in this experiment. Right: examples of calcium dynamics in individual ROIs in these brain areas. Black bar (top) indicates duration of stimulus (spots moving in rostro-caudal direction). Scale bar = 80 µm. The respective ROIs are highlighted in red.

Although we did not perform systematic measurements of neuronal activity in other brain areas, spontaneous activity was also observed in the forebrain and hindbrain. Figure 6 shows examples of fluorescence traces in the hindbrain (top) and the telencephalon (bottom). These areas did not respond to moving stimuli (shown on top as black bar) but showed prominent spontaneous activity. Thus, the *NBT:GCaMP3* line may also be useful for studying neuronal circuits in brain areas other than the optic tectum.

Taken together, these data demonstrate the potential of the *NBT:GCaMP3* line for imaging neuronal activity in late stage larvae.

## 4. Discussion

### 4.1. Imaging 2–3 Weeks Old Larvae

Previous studies have used zebrafish to study visually-driven behaviour in young zebrafish larvae and identify the underlying neuronal circuits. However, neuronal circuits regulating more complex behaviours, such as shoaling with conspecifics and shoaling preferences, have not been studied in detail. This is likely due to the fact that shoaling in zebrafish develops after 10–14 dpf [10,41,42], while the commonly used *huc* promoter-driven reporter lines decrease in fluorescence with age. Being the main visual area in lower vertebrates, the optic tectum is likely to be involved in the detection and localisation of moving conspecific fish. Therefore, it is important to image neuronal activity in the tectum in order to understand how these forms of behaviour develop and how they are regulated. To address this, we have generated a zebrafish line carrying a transgene comprising the *NBT* promoter driving strong transcription of GCaMP3 in neurons beyond 7–9 dpf (Figure 1). When imaged in an oxygenised solution, this transgenic line allows for robust imaging of the optic tectum in 14–21 dpf old larvae (Figure 3). We successfully applied this approach to image neuronal activity in the optic tectum and illustrate the usability of the approach by mapping the receptive fields of tectal neurons in 14–18 dpf larvae. A similar *NBT:GCaMP3* line was previously used to image neuronal activity in the habenula in 2–3 weeks old larvae [43].

In the past, three different approaches have been used to extend the period for imaging neuronal activity in zebrafish larvae. First, brighter forms of calcium reporters (e.g., GCaMP5) have been used instead of GCaMP3 (e.g., [17,18]). Second, calcium reporters have been targeted to the nucleus, using a UAS:gal4 system or a different pan-neuronal and/or specific promoter [12,14,16]. Finally, calcium dyes injected into the brain have also successfully been used to image adult zebrafish [13,44]. Each of these approaches has proven useful, within their own limitations. The choice of calcium indicator and the driving promoter will likely depend on the target brain area and specific research question. For example, calcium reporters fused to H2B and calcium dyes are not optimal to study the activity in synaptic layers, such as the tectal neuropil or the inner plexiform layer in the retina. *huc*-driven lines do not allow simultaneous imaging of presynaptic retinal input and postsynaptic activity in the tectal neuropil because HuC/D is expressed in retinal ganglion cells (Figure 2A). The *NBT:GCaMP3* line described here offers a complementary approach that may be particularly useful for studying activity in the tectal neuropil (Figure 2 and Figure 3).

Due to the scope of the project licences available for the research described here, we were unable to carry out experiments to assess neuronal activity in *NBT:GCAMP3* larvae beyond 21 dpf. In order to extend this period, it would be important to improve the oxygen supply to the fish. The ability of zebrafish larvae to take up oxygen through their skin decreases with age [45], and after 21 dpf, zebrafish larvae start to extensively breath through their gills. Therefore, increasing oxygen levels in the imaging solution is not sufficient anymore. An alternative approach of delivering oxygen is used in adult fish, and involves intubation with a small tube supplying water directly to the gills through the mouth. This approach has been used for adult zebrafish [13], and has recently been successfully applied to juvenile zebrafish (1 month post-fertilisation, [46]).

For the future, brighter alternatives to GCaMP3 (e.g., GCaMP6) or targeting of the calcium reporter to compartments adjacent to calcium channels (e.g., presynaptic terminals, [47]) could be used to further enhance the method described here.

### 4.2. RF Properties in Late Stage Larvae

Sajovic and Levinthal provided the first description of tectal receptive field properties in zebrafish [39]. Using small sweeping and flashing spots, they mapped RFs in the adult optic tectum and described four classes of tectal neurons, differing in their sensitivity to motion, direction of motion, spontaneous activity in the dark, and whole-field illumination. This research was followed up by Niell and Smith [38], who described tectal RFs in the developing visual system of zebrafish using larvae aged between 3 and 9 dpf. Their results largely confirmed the classification in four groups described by Sajovic and Levinthal, and spatial RF sizes seem to be slightly larger in larvae than in adult zebrafish (40 vs. 35 degrees respectively). Niell and Smith reported that most response properties emerge relatively early in development [38], and suggest that the tuning properties of tectal cells do not change drastically after 84 hpf. However, this seems to be not entirely true for all situations, as a more recent study, [48] describes that there is indeed an initial expansion and later refinement between 4 and 8 dpf, as has been described for many other sensory systems in the past. While it has been suggested that tectal RFs have reached maturity and do not change further after this refinement period, our data suggest that further refinement occurs beyond 9 dpf as RF size decreases with age.

## Supplementary Materials

The following are available online http://www.mdpi.com/2221-3759/6/1/6/s1. Figure S1: Imaging chamber and procedure of solution oxygenation; Figure S2: *NBT:GCaMP3* transgenic line: expression in the retina; Figure S3: Characterization of *NBT:GCaMP3* expression in the eye of 21 dpf fish.

## References

[1] Del Bene F., Wyart C., Robles E., Tran A., Looger L., Scott E.K., Isacoff E.Y., Baier H. (2010). Filtering of Visual Information in the Tectum by an Identified Neural Circuit. Science.

[2] Filosa A., Barker A.J., Maschio M.D., Baier H. (2016). Feeding State Modulates Behavioral Choice and Processing of Prey Stimuli in the Zebrafish Tectum. Neuron.

[3] Gabriel J.P., Trivedi C.A., Maurer C.M., Ryu S., Bollmann J.H. (2012). Layer-Specific Targeting of Direction-Selective Neurons in the Zebrafish Optic Tectum. Neuron.

[4] Preuss S.J., Trivedi C.A., Berg-Maurer C.M.V., Ryu S., Bollmann J.H. (2014). Classification of Object Size in Retinotectal Microcircuits. Curr. Biol..

[5] Roeser T., Baier H. (2003). Visuomotor Behaviors in Larval Zebrafish after Gfp-Guided Laser Ablation of the Optic Tectum. J. Neurosci..

[6] Severi K.E., Portugues R., Marques J.C., O’Malley D.M., Orger M.B., Engert F. (2014). Neural Control and Modulation of Swimming Speed in the Larval Zebrafish. Neuron.

[7] Thompson A.W., Vanwalleghem G.C., Heap L.A., Scott E.K. (2016). Functional Profiles of Visual-, Auditory-, and Water Flow-Responsive Neurons in the Zebrafish Tectum. Curr. Biol..

[8] Strahle U., Scholz S., Geisler R., Greiner P., Hollert H., Rastegar S., Schumacher A., Selderslaghs I., Weiss C., Witters H. (2012). Zebrafish Embryos as an Alternative to Animal Experiments-a Commentary on the Definition of the Onset of Protected Life Stages in Animal Welfare Regulations. Reprod. Toxicol..

[9] Andersson M.A., Ek F., Olsson R. (2015). Using Visual Lateralization to Model Learning and Memory in Zebrafish Larvae. Sci. Rep..

[10] Dreosti E., Lopes G., Kampff A.R., Wilson S.W. (2015). Development of Social Behavior in Young Zebrafish. Front. Neural Circuits.

[11] Valente A., Huang K.H., Portugues R., Engert F. (2012). Ontogeny of Classical and Operant Learning Behaviors in Zebrafish. Learn. Mem..

[12] Kim C.K., Miri A., Leung L.C., Berndt A., Mourrain P., Tank D.W., Burdine R.D. (2014). Prolonged, Brain-Wide Expression of Nuclear-Localized Gcamp3 for Functional Circuit Mapping. Front. Neural Circuits.

[13] Kassing V., Engelmann J., Kurtz R. (2013). Monitoring of Single-Cell Responses in the Optic Tectum of Adult Zebrafish with Dextran-Coupled Calcium Dyes Delivered Via Local Electroporation. PLoS ONE.

[14] Nikolaev A., Leung K.M., Odermatt B., Lagnado L. (2013). Synaptic Mechanisms of Adaptation and Sensitization in the Retina. Nat. Neurosci..

[15] Nikolaou N., Lowe A.S., Walker A.S., Abbas F., Hunter P.R., Thompson I.D., Meyer M.P. (2012). Parametric Functional Maps of Visual Inputs to the Tectum. Neuron.

[16] Odermatt B., Nikolaev A., Lagnado L. (2012). Encoding of Luminance and Contrast by Linear and Nonlinear Synapses in the Retina. Neuron.

[17] Jetti S.K., Vendrell-Llopis N., Yaksi E. (2014). Spontaneous Activity Governs Olfactory Representations in Spatially Organized Habenular Microcircuits. Curr. Biol..

[18] Matsuda K., Yoshida M., Kawakami K., Hibi M., Shimizu T. (2017). Granule Cells Control Recovery from Classical Conditioned Fear Responses in the Zebrafish Cerebellum. Sci. Rep..

[19] Vendrell-Llopis N., Yaksi E. (2015). Evolutionary Conserved Brainstem Circuits Encode Category, Concentration and Mixtures of Taste. Sci. Rep..

[20] Burgess H.A., Schoch H., Granato M. (2010). Distinct Retinal Pathways Drive Spatial Orientation Behaviors in Zebrafish Navigation. Curr. Biol..

[21] Bianco I.H., Engert F. (2015). Visuomotor Transformations Underlying Hunting Behavior in Zebrafish. Curr. Biol..

[22] Muto A., Ohkura M., Abe G., Nakai J., Kawakami K. (2013). Real-Time Visualization of Neuronal Activity During Perception. Curr. Biol..

[23] Dunn T.W., Gebhardt C., Naumann E.A., Riegler C., Ahrens M.B., Engert F., del Bene F. (2016). Neural Circuits Underlying Visually Evoked Escapes in Larval Zebrafish. Neuron.

[24] Temizer I., Donovan J.C., Baier H., Semmelhack J.L. (2015). A Visual Pathway for Looming-Evoked Escape in Larval Zebrafish. Curr. Biol..

[25] Hunter P.R., Lowe A.S., Thompson I.D., Meyer M.P. (2013). Emergent Properties of the Optic Tectum Revealed by Population Analysis of Direction and Orientation Selectivity. J. Neurosci..

[26] Bronchain O.J., Hartley K.O., Amaya E. (1999). A Gene Trap Approach in Xenopus. Curr. Biol..

[27] Tian L., Hires S.A., Mao T., Huber D., Chiappe M.E., Chalasani S.H., Petreanu L., Akerboom J., McKinney S.A., Schreiter E.R. (2009). Imaging Neural Activity in Worms, Flies and Mice with Improved Gcamp Calcium Indicators. Nat. Methods.

[28] Kawakami K. (2005). Transposon Tools and Methods in Zebrafish. Dev. Dyn..

[29] Kawakami K. (2007). Tol2: A Versatile Gene Transfer Vector in Vertebrates. Genome Biol..

[30] Suster M.L., Sumiyama K., Kawakami K. (2009). Transposon-Mediated Bac Transgenesis in Zebrafish and Mice. BMC Genom..

[31] Balciunas D., Wangensteen K.J., Wilber A., Bell J., Geurts A., Sivasubbu S., Wang X., Hackett P.B., Largaespada D.A., McIvor R.S. (2006). Harnessing a High Cargo-Capacity Transposon for Genetic Applications in Vertebrates. PLoS Genet..

[32] Lister J.A., Robertson C.P., Lepage T., Johnson S.L., Raible D.W. (1999). Nacre Encodes a Zebrafish Microphthalmia-Related Protein That Regulates Neural-Crest-Derived Pigment Cell Fate. Development.

[33] Brainard D.H. (1997). The Psychophysics Toolbox. Spat. Vis..

[34] Kleiner M., Brainard D., Pelli D. (2007). What’s New in Psychtoolbox-3?. Perception.

[35] Pelli D.G. (1997). The Videotoolbox Software for Visual Psychophysics: Transforming Numbers into Movies. Spat. Vis..

[36] Dorostkar M.M., Dreosti E., Odermatt B., Lagnado L. (2010). Computational Processing of Optical Measurements of Neuronal and Synaptic Activity in Networks. J. Neurosci. Methods.

[37] Thevenaz P., Ruttimann U.E., Unser M. (1998). A Pyramid Approach to Subpixel Registration Based on Intensity. IEEE Trans. Image Process..

[38] Niell C.M., Smith S.J. (2005). Functional Imaging Reveals Rapid Development of Visual Response Properties in the Zebrafish Tectum. Neuron.

[39] Sajovic P., Levinthal C. (1982). Visual Cells of Zebrafish Optic Tectum: Mapping with Small Spots. Neuroscience.

[40] Walker A.S., Burrone J., Meyer M.P. (2013). Functional Imaging in the Zebrafish Retinotectal System Using Rgeco. Front. Neural Circuits.

[41] Hinz R.C., de Polavieja G.G. (2017). Ontogeny of Collective Behavior Reveals a Simple Attraction Rule. Proc. Natl. Acad. Sci. USA.

[42] Buske C., Gerlai R. (2011). Shoaling Develops with Age in Zebrafish (*Danio rerio*). Prog. Neuropsychopharmacol. Biol. Psychiatry.

[43] Krishnan S., Mathuru A.S., Kibat C., Rahman M., Lupton C.E., Stewart J., Claridge-Chang A., Yen S.C., Jesuthasan S. (2014). The Right Dorsal Habenula Limits Attraction to an Odor in Zebrafish. Curr. Biol..

[44] Hollmann V., Lucks V., Kurtz R., Engelmann J. (2015). Adaptation-Induced Modification of Motion Selectivity Tuning in Visual Tectal Neurons of Adult Zebrafish. J. Neurophysiol..

[45] Rombough P., Drader H. (2009). Hemoglobin Enhances Oxygen Uptake in Larval Zebrafish (*Danio rerio*) but Only under Conditions of Extreme Hypoxia. J. Exp. Biol..

[46] Olt J., Allen C.E., Marcotti W. (2016). In Vivo Physiological Recording from the Lateral Line of Juvenile Zebrafish. J. Physiol..

[47] Dreosti E., Odermatt B., Dorostkar M.M., Lagnado L. (2009). A Genetically Encoded Reporter of Synaptic Activity in Vivo. Nat. Methods.

[48] Zhang M., Liu Y., Wang S.Z., Zhong W., Liu B.H., Tao H.W. (2011). Functional Elimination of Excitatory Feedforward Inputs Underlies Developmental Refinement of Visual Receptive Fields in Zebrafish. J. Neurosci..

