# Supplementary material for: Imaging Neuronal Activity in the Optic Tectum of Late Stage Larval Zebrafish
Source: J Dev Biol. 2018 Mar 9;6(1):6. doi: 10.3390/jdb6010006 (PMC5875565; doi:10.3390/jdb6010006)
Supplement: Supplementary file 1 [file jdb-06-00006-s001.pdf]

Journal of Developmental Biology

MDPI

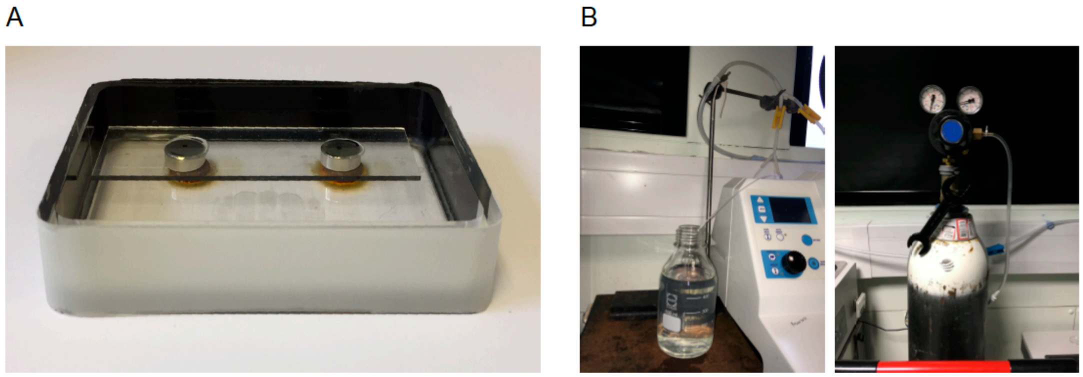

Supplementary Figure 1. Imaging chamber and the procedure of solution oxygenation. **(A)** Fish was imaged in a plastic custom-made chamber (90x70x15 mm). Three sides of the chamber were covered with black tape and the screen was covered with an opaque film (See Methods for more information). **(B)** Oxygenation was delivered through plastic tubing (left) from a gas bottle containing O_2/CO_2 mix (right).

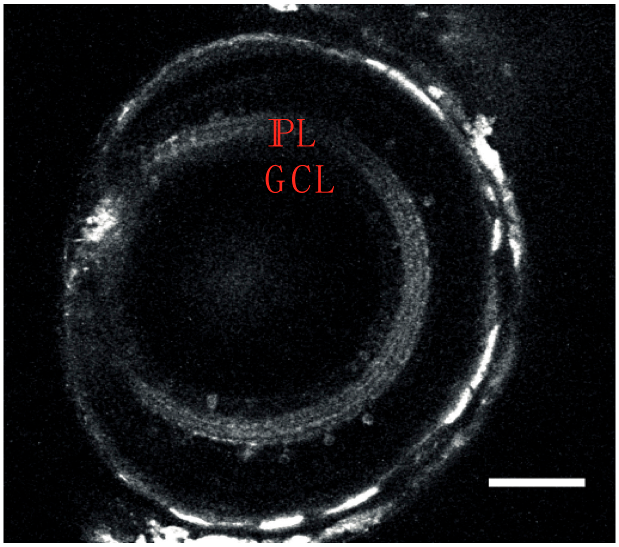

Supplementary Figure 2. NBT:GCaMP3 transgenic line; expression in the retina (right, 10 dpf). In the eye, GCaMP3 is expressed in the inner plexiform layer (depicted as IPL) and in a small number of cells (2.75 ± 0.9 per field of view, n = 10 fields of view) in the ganglion cell layer (depicted as GCL). For presentation purposes, images were filtered using a Gaussian filter (sigma = 1).

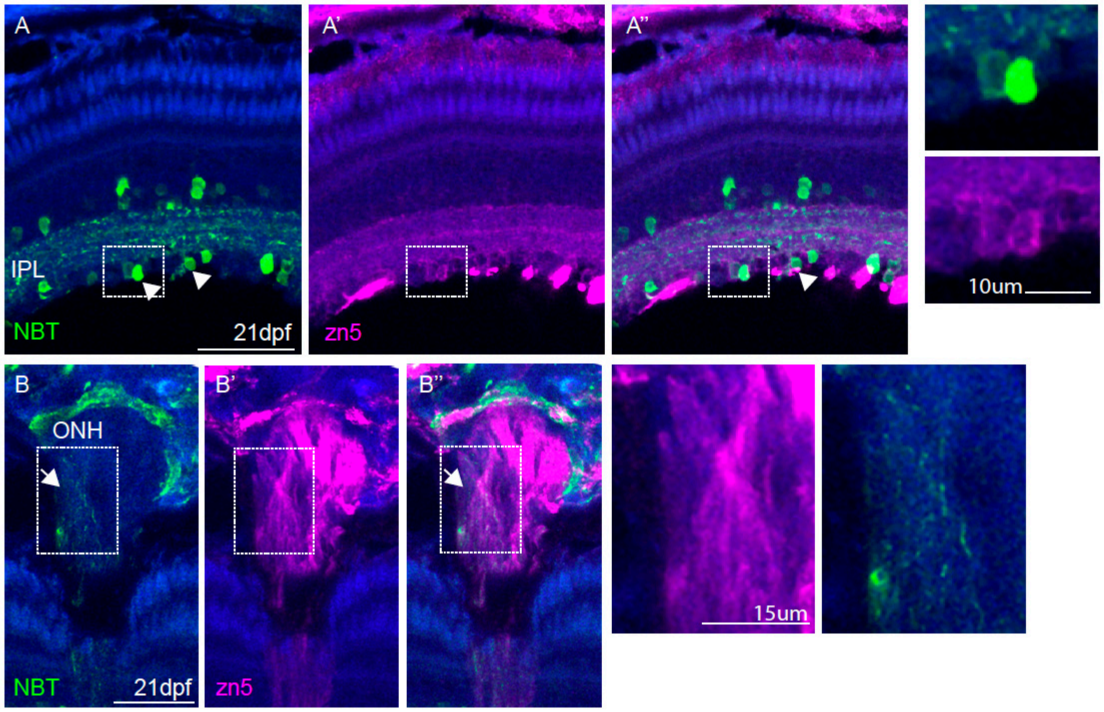

Supplementary Figure 3. Characterisation of NBT:GCaMP3 expression in the eye of 21 dpf fish. A, a small number of neurons in the ganglion cell layer (arrowheads) are zn5 positive, a marker for retinal ganglion cells. The inset on the right shows labelling of two individual cells in the ganglion cell layer. B, the NBT promoter labels a small number of retinal ganglion cell axons in the optic nerve (ONH, white arrows). The blue label is a DAPI stain. Scale bars in A=50μm, B = 25μm
